# Modulation of epileptogenesis through transplantation of human mesenchymal stem cells with or without GDNF release

**DOI:** 10.1007/s00018-025-05853-z

**Published:** 2025-08-21

**Authors:** Eliška Waloschková, Esbjörn Melin, Camille Baumlin, My Andersson, Alberto Martínez Serrano, Merab Kokaia, Marco Ledri

**Affiliations:** 1 Experimental Epilepsy Group, Epilepsy Center, Department of Clinical Sciences, Lund University Hospital, Lund, 22184 Sweden; 2 Department of Molecular Biology, Department of Molecular Neuropathology, Center of Molecular Biology Severo Ochoa (UAM-CSIC), Universidad Autónoma de Madrid, Nicolás Cabrera 1, Madrid, 28049 Spain; 3 Cellular Neurophysiology and Epilepsy Group, Epilepsy Center, Department of Clinical Sciences, Lund University Hospital, Lund, 22184 Sweden; 4 Molecular Neurophysiology and Epilepsy Group, Epilepsy Center, Department of Clinical Sciences, Lund University Hospital, Epilepsy Center, Lund, 22184 Sweden

**Keywords:** Human mesenchymal stem cells, GDNF, Epileptogenesis, Epilepsy, Inflammation

## Abstract

Epilepsy is a central nervous system disorder causing uncontrollable seizures. One-third of patients do not respond to current medications, necessitating new treatments. This study targeted epileptogenesis, the process leading to chronic epilepsy, using human mesenchymal stem cells (MSCs) in a rodent model. MSC transplantation can positively affect neurodegenerative diseases by modifying inflammation. Additionally, glial cell line-derived neurotrophic factor (GDNF) may counteract seizures and tissue damage. We transplanted naïve immortalized human adipose-derived MSCs (Ctrl-MSCs) or GDNF-releasing MSCs (GDNF-MSCs, releasing 588.67 ± 20.14 pg/ml/24 h GDNF) into rat hippocampi after kainic acid-induced status epilepticus. Seizure progression was monitored for 5 weeks using video-EEG, behavioral assessments, and histological analysis. Both cell types influenced epileptogenesis. GDNF-MSCs delayed early-stage seizures, while Ctrl-MSCs reduced seizure frequency in later stages. Differences emerged in seizure development and cumulative seizure count, with Ctrl-MSCs showing significant seizure-attenuating effects. Behavioral differences were also noted: Ctrl-MSCs improved short-term memory and reduced anxiety, whereas GDNF-MSCs primarily reduced anxiety without significantly improving memory. This study highlights the therapeutic potential of MSCs, with or without GDNF, in modulating epileptogenesis, offering promising avenues for future clinical treatments.

**Supplementary Information:**

The online version contains supplementary material available at 10.1007/s00018-025-05853-z.

## Background

Epilepsy is a chronic condition affecting the central nervous system (CNS) and is characterized by spontaneously recurring seizures (SRSs). It is considered to be the second most burdensome neurological disorder in terms of disability-adjusted life years [1] and affects approximately 7 out of 1000 people worldwide [2]. Notably, anti-seizure medications (ASMs) fail to suppress seizures in approximately one-third of epilepsy patients. One of the most prevalent forms of such drug-resistant epilepsy is temporal lobe epilepsy (TLE) [3]. The chronic stage of this disorder is thought to result from an initial brain insult followed by a latent period, during which epileptogenesis occurs [4]. Epileptogenesis involves a cascade of molecular, cellular and network alterations that ultimately result in the development of SRSs. The initial damaging event can stem from various sources, including ischemic stroke, traumatic brain injury, cortical dysplasia, tumors, or prolonged acute symptomatic seizures, such as status epilepticus (SE) [5]. Therefore, preventive therapies could be implemented if individuals at risk of developing chronic epilepsy could be identified through reliable biomarkers. However, there are currently no prophylactic treatments available to prevent the onset of epilepsy in such patients.

The cellular and molecular changes that occur during epileptogenesis include, among others, neurodegeneration, activation of inflammatory pathways, gliosis or axonal injury and sprouting [6]. This process is most thoroughly characterized in the hippocampus of SE models of TLE, where a latency period of days to weeks exists between SE induction and the appearance of the first SRSs [7]. Intervening therapeutically during the late phase of SE, or shortly thereafter, could offer a promising strategy for preventing or mitigating the severity of epilepsy in its later chronic phase, potentially altering disease progression [5]. Various studies have reported the beneficial effects of transplanting mesenchymal stem cells (MSCs) for therapeutic purposes [8–14]. MSCs have neuroprotective and anti-inflammatory properties in several CNS disorders, including stroke [15], multiple sclerosis [16], spinal cord injury [17] and Parkinson’s disease [18]. Their safety and efficacy are currently being evaluated in numerous clinical trials [19]. The positive outcomes of MSC transplantation are believed to stem from their paracrine mechanisms, which involve the release of various cytokines and growth factors. These factors help modulate the immune response, promote angiogenesis and neurogenesis, and regulate neuronal functions [20–23]. Indeed, neurotrophins have emerged as potential targets for epilepsy treatment. Although their effects in this context have been reported with some variability [24], accumulating evidence indicates that specific neurotrophic factors, such as glial cell line-derived neurotrophic factor (GDNF), could be effective in counteracting seizures and reducing neuronal damage in animal models. One study showed that recombinant adeno-associated virus (rAAV) vector-based GDNF overexpression in the rat hippocampus suppressed seizure activity in models of temporal lobe epilepsy [25]. A different approach involving the implantation of encapsulated cells that release GDNF in the hippocampus has been shown to effectively reduce seizures in various rodent models [26–28]. Moreover, GDNF signaling is thought to alter the epileptogenesis process, delaying seizure onset and reducing neurodegeneration in a mouse TLE model [29]. In this study, we combined these two therapeutic strategies by utilizing either naïve human immortalized adipose-derived MSCs (hiAd-MSCs; Ctrl-MSCs) or hiAd-MSCs genetically engineered to release GDNF (GDNF-MSCs) to target and influence the process of epileptogenesis. We transplanted these cells into the hippocampi of rats 16–24 h after kainic acid (KA)-induced SE and conducted continuous wireless video-EEG monitoring for 35 days. Following the monitoring period, the animals were subjected to tests to assess anxiety, locomotion, and memory. Finally, the tissue samples were collected for comprehensive histopathological analysis. These results indicate that treatment with MSCs lacking GDNF release exhibited superior beneficial effects in counteracting the process of epileptogenesis compared to MSCs engineered to release GDNF.

## Materials and methods

The work has been reported in line with the ARRIVE guidelines 2.0.

### Animals

Male Sprague Dawley rats (Janvier Labs, Le Genest-Saint-Isle, France) were housed in individually ventilated cages under a 12/12-h light cycle with *ad libitum* access to food and water. A total of 94 rats were used.

After experiments were concluded, the animals were anesthetized with an overdose of pentobarbital and transcardially perfused first with 0.9% ice-cold saline and subsequently with 4% paraformaldehyde (PFA) in 0.1 M phosphate buffer (PB). Dissected brains were postfixed in 4% PFA overnight at 4 °C, transferred to a 25% sucrose solution and stored at 4 °C. After at least 3 days in sucrose, the brains were horizontally sectioned on a microtome into 30 µm slices and kept in a glycerol-based antifreeze solution at −20 °C until further processing.

### Cell culture

Human adipose-derived mesenchymal stem cells (hiAD-MSCs; ASC52telo, hTERT immortalized mesenchymal stem cells; ATCC SCRC4000, ATCC, Manassas, VA, USA) were used in this study. The two cell lines generated were hiAD-mCh-MSCs (expressing mCherry) and hiAD-GDNF-mCh-MSCs (expressing GDNF and mCherry). Briefly, naïve hiAD-MSCs were transduced with lentiviral particles containing either pCCL_EF1a-mCh or pCCL_EF1a-GDNF-mCh and sorted by flow cytometry on the basis of intense mCherry fluorescence. The plasmids were generated by Cristina Salado Manzano at CIEMAT (Madrid, Spain) and donated by Dr. Josep M. Canals Coll from Universitat de Barcelona. The viral particles were produced via the Viral Vector service of Centro Nacional de Investigaciones Cardiovasculares (Madrid, Spain) by cotransfecting 293 T cells with a lentiviral plasmid and a packaging plasmid. Viral titers were obtained as physical titers in physical particles (PP)/mL by performing qPCR on the viral genomes with primers against LTRs. The MOI for transfection was calculated considering a ratio of PP/transduction units (TUs) equal to 100. For lentiviral transduction, MSCs were seeded in 12-well plates at a density of 7000 cells/cm^2^ and transduced in proliferation medium containing 4 µg/mL polybrene for 24 h. After 3 passages, the cells were collected, FACS-sorted, reseeded, expanded and cryopreserved at passage 7. For FACS, the transduced cells were excited with a yellow‒green laser at 561 nm, and mCherry fluorescence emission was detected by a 610/20 nm detector via a FACSAria Fusion system (BD Biosciences, Franklin Lakes, NJ). The cells were cultured in T75 flasks in Mesenchymal Stem Cell Basal Medium (ATCC PCS-500-030, ATCC, Manassas, VA, USA) supplemented with a Mesenchymal Stem Cell Growth Kit (ATCC PCS-500-040, ATCC, Manassas, VA, USA), penicillin‒streptomycin (10 units/ml, Gibco, Waltham, MA, USA) and amphotericin B (25 ng/ml, Gibco, Waltham, MA, USA). The media was changed once every 3–4 days. The cells were expanded as needed and cryopreserved. The same passage was used for all in vivo experiments.

### Cell quality assessment

After cell transplantation, the remaining cells were replated in 12-well plates (~ 13,000 cells/well) in the same culture media. After 3 days in culture, the media was collected for ELISA measurements and replaced with differentiation media (StemPro Adipogenesis Differentiation Kit or StemPro Osteogenesis Differentiation Kit, both from Gibco, Waltham, MA, USA). The media was replaced every 3–4 days. After 21 days of differentiation, the cells were fixed with 4% PFA in phosphate buffer for 30 min. The analysis revealed that 93.2% of cells were positive for Oil Red staining and 72.5% were positive for Azarin Red staining.

### Status epilepticus induction

Male Sprague–Dawley rats (7–8 weeks old) were injected subcutaneously in the neck region with an initial dose of 5 mg/kg KA and subsequently with 2.5 mg/kg KA every hour until the first stage 3 or higher seizure grade was observed. Seizures were classified according to the modified Racine scale registering only stages 3 and higher: (3) unilateral forelimb clonus; (4) generalized seizure with rearing, body jerks, or bilateral forelimb clonus; and (5) generalized seizure with rearing, imbalance, falling or wild running [30]. SE was defined as at least 4 seizures per hour. After SE, the animals were injected with a Ringer/glucose (25 mg/ml) solution (1:1 ratio) and returned to the housing cages. Cell transplantation was performed 16–24 h after SE induction.

### Cell transplantation and electrode implantation

The cells were thawed and, after 2 days in culture, detached with TrypLE™ Express Enzyme (Gibco, Waltham, MA, USA). The cells were then centrifuged and resuspended in HBSS (Gibco, Waltham, MA, USA) containing DNase I solution (1 mg/ml; Stem Cell Technologies, Vancouver, BC, Canada) to a concentration of 100,000 cells/ml and kept on ice until transplantation. For surgeries, the rats were anesthetized with 4% isoflurane, placed in a stereotaxic frame and maintained on 2% isoflurane. The cells were then stereotaxically injected bilaterally into both hippocampi at the following coordinates (from bregma): anterior-posterior − 5.6 mm, medial-lateral ± 5.0 mm, dorsal-ventral − 6.0, −4.8 and − 3.6 mm, for a total of 3 µl per hippocampus (1 µl at each deposit site). After cell injections, electrodes and transmitters for wireless video-EEG monitoring were implanted. The procedure was performed as described previously [31]. The transmitter (F40-EET, Data Sciences International, St. Paul, MN, USA) was inserted in a subcutaneous pocket on the backs of the rats. One stainless steel electrode (Plastics One, Roanoke, VA, USA), soldered to the wire of the transmitter, was implanted at the same coordinates as above in the right hemisphere. The second electrode was placed on top of the dura mater above the motor cortex ipsilateral to the depth electrode. Two reference electrodes were placed on the dura mater, 2 mm rostral to the lambda. One stainless steel screw was attached to the skull bone to secure the electrode assembly with dental cement. The animals were then injected with a Ringer/glucose (25 mg/ml) solution (1:1 ratio) and returned to the housing cages. The animals were weighed every day for one week after implantation and, consequently, once per week. To begin video-EEG monitoring, the cage was placed on top of a receiver unit (Data Sciences International, St. Paul, MN, USA), and the wireless transmitter was activated by a magnet. Four cameras (Axis, Lund, Sweden) were used to record videos of animal activity continuously for 35 days, and seizures were then detected offline in NeuroScore (Data Sciences International, St. Paul, MN, USA). A cohort of animals was used for tissue collection at 7, 14 and 21 days after transplantation.

### Behavioral testing

After 35 days of video-EEG monitoring, the rats were subjected to behavioral tests. ANY-maze software (Stoelting, Wood Dale, IL, USA) was used to perform and analyze all the tests. Open field arenas (1 × 1 m) were used for anxiety and locomotion measurements, where the first 15 min of the open field test were analyzed for anxiety-related behavior and the last 15 min were analyzed for locomotion. The next day, a simple novel object recognition test was performed in the same arenas. The animals were placed in arenas containing 2 identical objects in the learning phase for 15 min. After a 3-h break, the animals were placed back into the same arena, with one of the 2 objects being exchanged for a novel object. The animals were monitored in this recognition phase for 15 min.

### Immunocytochemistry, immunohistochemistry and image analysis

For immunocytochemistry, the fixed cells were thoroughly washed with distilled water 2 times. For osteocyte staining, the cells were incubated with 2% Alizarin Red Staining Solution (Sigma Aldrich, St. Louis, MO, USA) for 2–3 min and then rinsed well with water 3 times. For adipocyte staining, the cells were incubated with 60% isopropanol for 5 min, incubated with Oil Red O solution (Sigma Aldrich, St. Louis, MO, USA) for 15 min and washed 5 times with water. Images were acquired on an inverted microscope (Olympus CKX53, Olympus, Shinjuku, Tokyo, Japan).

For immunohistochemistry, the rats were sacrificed at 7, 14, 21 or 40 days after cell transplantation. For staining, the sections were washed thoroughly with phosphate-buffered saline (PBS), blocked in 5% serum specific to the secondary antibody in PBS containing 0.25% Triton-X and incubated with the primary antibody (1:200, CD68 antibody/ED1, MCA341R, Bio-Rad, Hercules, CA, USA/1:2000, anti-Cherry antibody, ab205402, Abcam, Cambridge, UK) overnight in the same solution at 4 °C. Following primary antibody incubation, the sections were washed with PBS and blocked again with the same serum mixture described above. Then, the sections were incubated with fluorophore-conjugated secondary antibodies for 2 h (AlexaFluor Plus 488/555, 1:1000, Thermo Fisher, Waltham, MA, USA), washed thoroughly with PBS, incubated with 1:1000 Hoechst in PBS solution and coverslipped with PVA-DABCO.

Images were acquired via epifluorescence microscopy (Olympus BX61, Olympus, Shinjuku, Tokyo, Japan). For quantification, ImageJ software (NIH, Annapolis, MD, USA) was used. The mean fluorescence intensity was measured in images taken at 20x magnification. Images were taken within 3 sections throughout the dorsal‒ventral axis in each rat (5 rats per group were used), with 3 images per hippocampus (CA1, CA3 and dentate gyrus). The mean fluorescence intensity values were background-corrected and averaged from all images per animal. Averaged values were used for statistical analysis.

### ELISA

The amount of GDNF released from Ctrl-MSCs and GDNF-MSCs was examined after replating the remaining cells after transplantation surgeries. The cells were cultured for 3 days, and media samples were collected to quantify GDNF release via a commercially available kit (GDNF Human ELISA Kit, Invitrogen, Waltham, MA, USA). The results are presented as ng/ml/GDNF per 24 h.

### Statistical analyses

Statistical analysis of all the data were performed via Prism 9 software (GraphPad, San Diego, CA, USA). For normally distributed data, an unpaired t test was used; when the normality of the data was not confirmed, a Mann‒Whitney test was used instead. For paired data, the Wilcoxon test was used, and for comparisons of proportions, the Chi-squared **χ²** test was used. The Kruskal‒Wallis test was used for comparing more than two groups together with Dunn’s multiple comparisons test. Linear or nonlinear regression was used to compare the data with the fitted curves. The level of significance for these tests was set at *p* < 0.05. The Kolmogorov‒Smirnov test was used for distribution comparisons, and the level of significance was set to *p* < 0.01 and D > 0.1. The mean ± SEM is used to represent values in the main text; in graphs, either the mean ± SEM is shown or the median with the interquartile range in the case of nonnormally distributed data.

## Results

### hiAd-MSCs retain their properties in vitro and survive after transplantation in vivo

**Fig. 1:**
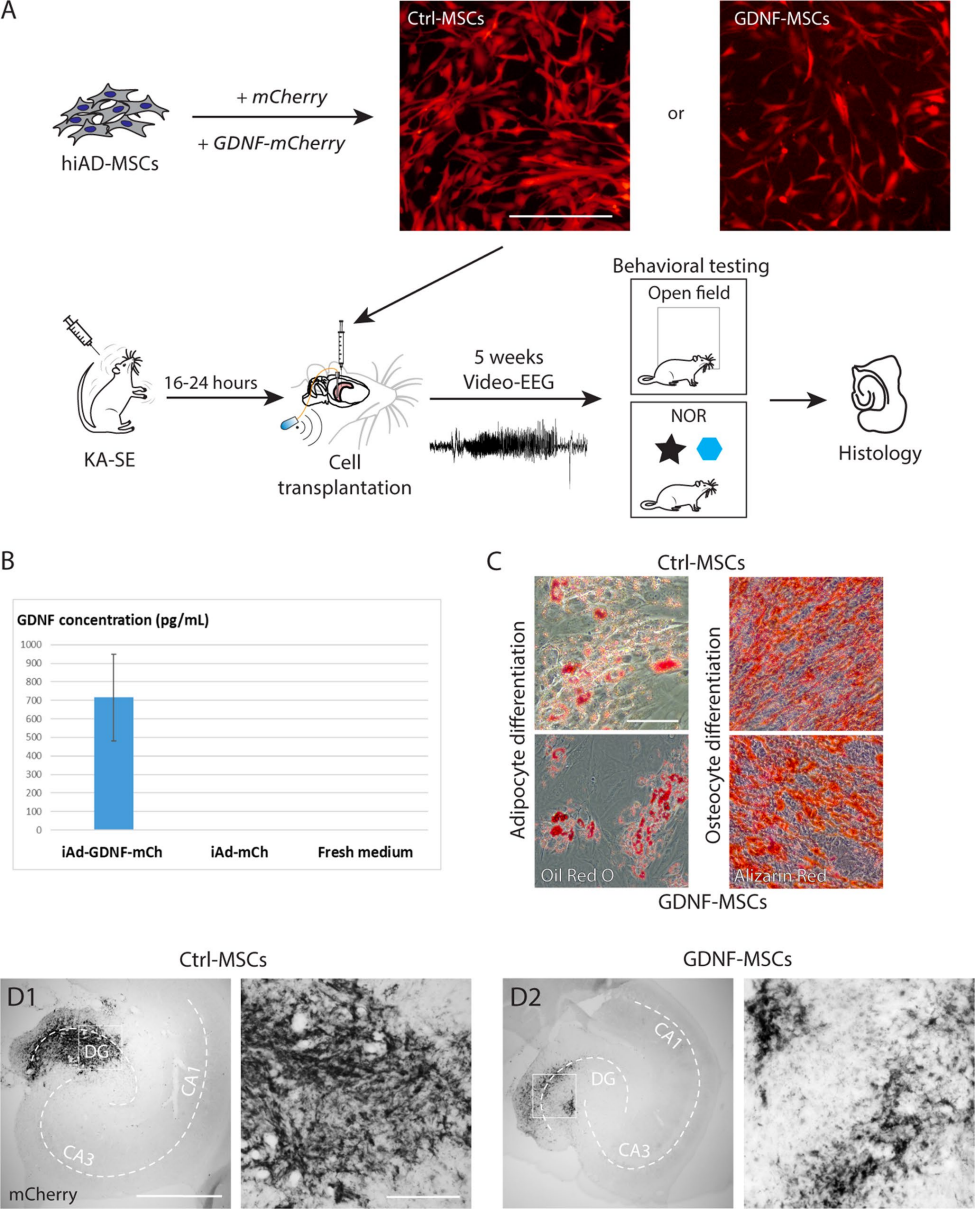
Experimental timeline and cell quality assessment. (**A**) Schematic illustration of the study timeline, including visualization of mCherry expression in Ctrl-MSCs and GDNF-MSCs in vitro. (**B**) GDNF release from GDNF-transfected cells and controls. (**C**) Post-transplantation differentiation of both cell lines in vitro into adipocytes (Oil Red O staining) and osteocytes (Alizarin Red staining) confirmed the persistent multipotency of the MSCs. (**D**) Images of the rat hippocampus stained with mCherry 7 days after MSC transplantation, confirming the survival of Ctrl-MSCs (D1) and GDNF-MSCs (D2) in vivo. Scale bars: 1 mm and 200 mm

**Fig. 2:**
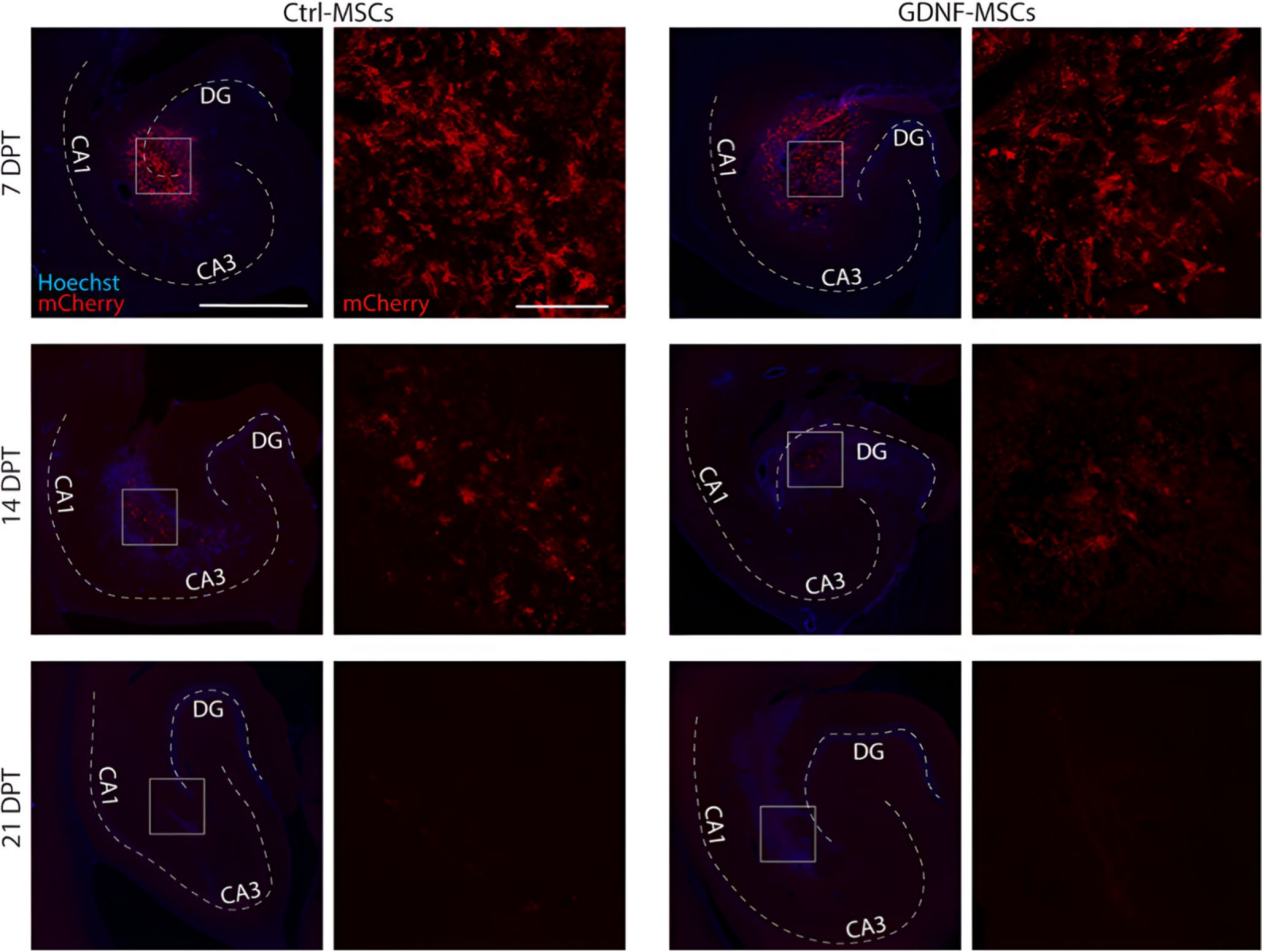
Cell survival after transplantation in vivo. The rat hippocampus was stained with mCherry at 7, 14 and 21 days after MSC transplantation, confirming the survival of Ctrl-MSCs and GDNF-MSCs in vivo until 14 days. Scale bars: 1 mm and 200 mm

The mCherry expression of the hiAd-MSCs was first assessed in vitro prior to transplantation (Fig. 1A). The cells were then transplanted bilaterally into the hippocampi of rats 16–24 h after SE (Fig. 1A). After transplantation surgeries, the remaining leftover cells were replated, and 3 days later, the media was collected to assess the levels of GDNF release from both cell lines. ELISA measurements confirmed the release of GDNF from GDNF-MSCs (588.67 ± 20.14 pg/ml/24 h; *n* = 4) but not from Ctrl-MSCs (values undetectable). Additionally, after media collection, both cell lines were differentiated into either adipocytes or osteocytes to verify their multipotency. Both Ctrl-MSCs and GDNF-MSCs were routinely able to differentiate into these two lineages, as confirmed by Oil Red O staining (for adipocytes) and Alizarin Red staining (for osteocytes) after 21 days of differentiation (Fig. 1B). To confirm the grafting ability and examine the survival of the cells in vivo, a cohort of rats was used for immunohistochemistry at 7, 14 and 21 days after cell transplantation. Surviving cells were observed in the hippocampi of all animals at 7 days and 14 days after transplantation, whereas at 21 days, no remaining cells were found. (Fig. 2).

### hiAd-MSC transplantation attenuates seizure occurrence during epileptogenesis

**Fig. 3:**
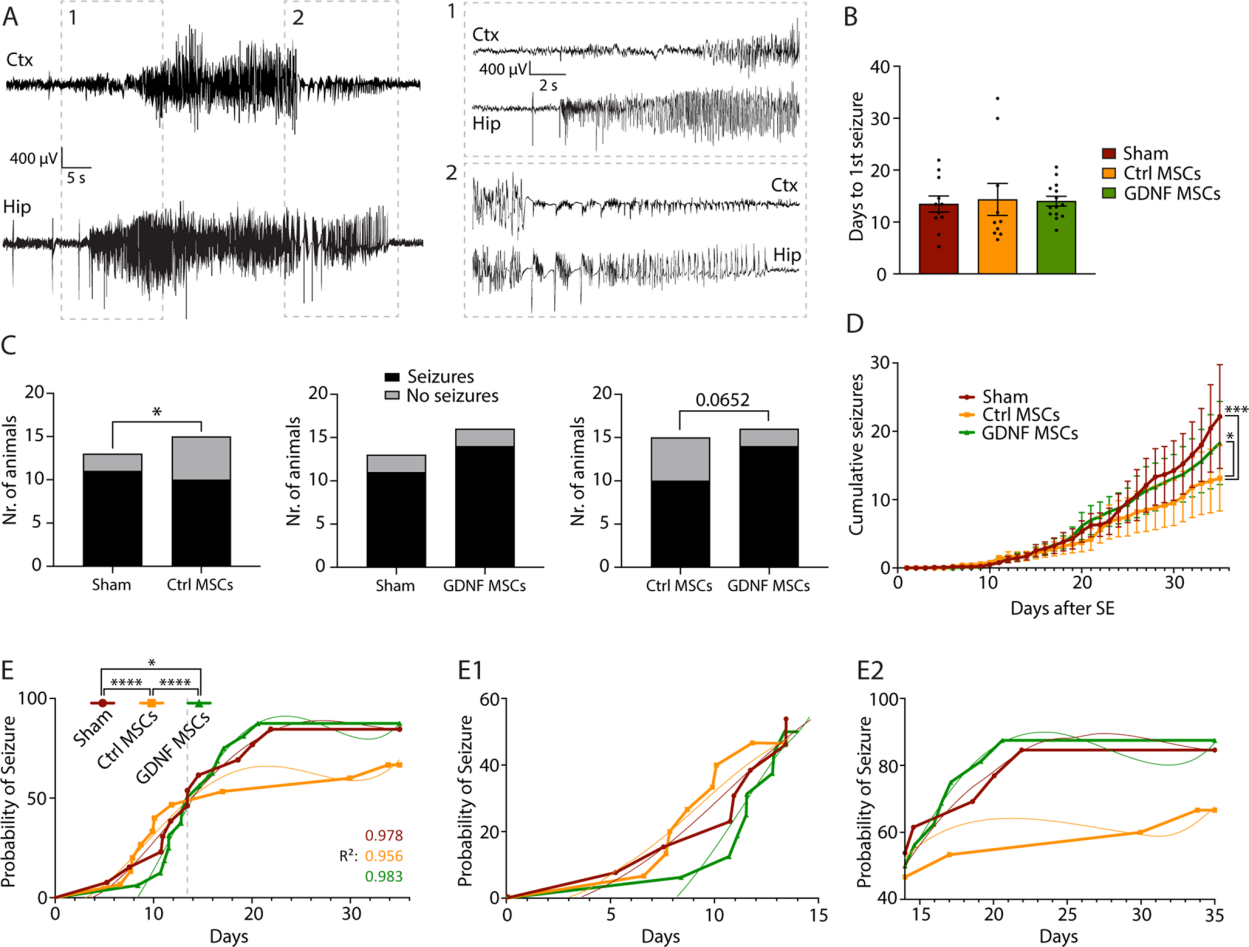
Effect of hiAd-MSCs on the development of SRS. (**A**) Representative EEG traces of a seizure. Seizure activity starts in the hippocampus, propagates into the motor cortex (zoom-in 1), and then returns to normal EEG activity (zoom-in 2). (**B**) Cumulative number of seizures during 35 days of video-EEG monitoring compared between the sham, Ctrl-MSC-treated and GDNF-MSC-treated groups. Linear regression revealed significant differences between the sham and Ctrl-MSC groups but not between the sham and GNDF-MSC groups. No statistically significant differences were detected between the individual data points. (**C**) Comparison of the proportions of animals that developed SRSs during the 35 days of monitoring. Fewer animals developed SRSs in the Ctrl-MSC-treated group than in the sham group. GDNF-MSCs had no effect. (**D**) Neither cell line had a significant effect on the onset of SRSs, represented as days to first seizure. (**E**) Probability of seizures depicted as survival curves for each group. When polynomial curves were fitted and nonlinear regression was performed, differences were observed between all 3 groups. (E1) Zoomed-in image of the first 2 weeks after cell transplantation, where the effect of GDNF-MSCs can be observed. (E2) Zoomed-in images of the last 3 weeks of monitoring, where the GDNF-MSCs had no effect, while the Ctrl-MSCs differed from those in the other 2 groups. Linear regression was used for comparisons in B. The c^2^ test was used for comparing proportions in C. The Kruskal‒Wallis test with Dunn’s multiple comparisons test was used in D, and nonlinear regression was used for curve comparisons in E. *, *p* < 05; ***, *p* < 001; ****, *p* < 0001

The occurrence of SRSs was investigated by continuous video-EEG monitoring during the epileptogenesis period for 35 days after cell transplantation (i.e., until 36 days after SE induction). All animals included in these experiments survived SE, however not all animals developed SRSs (Fig. 3C). Seizures predominantly originated in the hippocampus, and in 97.1% of cases, they propagated into the motor cortex (736 out of 758 total seizures; representative EEG traces are depicted in Fig. 3A). Cumulative seizure analysis via simple linear regression revealed a decrease in the seizure occurrence rate, with statistically significant differences between the Sham (*n* = 13) and Ctrl-MSC-treated animals (*n* = 15; F = 12.18, *p* = 0.0005) and between the Ctrl-MSC- and GDNF-MSC-treated animals (*n* = 16; F = 6.539, *p* = 0.0107), but interestingly, not between the Sham and GDNF-MSC-treated animals (F = 1.265, *p* = 0.2610; Fig. 3B). The Ctrl-MSCs also decreased the number of animals that developed SRSs during the 35 days of monitoring. When the proportions of animals that developed seizures and those that did not develop seizures were compared, a statistically significant difference was observed between the Sham and Ctrl-MSC groups (Sham: 2 out of 13 not showing SRSs; Ctrl-MSCs: 5 out of 15 not showing SRSs; *p* = 0.0321). However, no significant differences were detected between the sham and GDNF-MSC groups (GDNF-MSCs: 2 out of 16 not showing SRSs; *p* = 0.3657). Notably, the onset of seizures (days to the first seizure) did not differ between the groups (sham: 13.48 ± 1.54 days; Ctrl-MSCs: 14.35 ± 3.08 days; GDNF-MSCs: 14.02 ± 0.93 days; *p* = 0.4671; Fig. 3D). Survival analysis of the probability of seizure occurrence did not reveal differences between the groups (*p* = 0.5653). However, when polynomial curves were fitted through the data points and nonlinear regression was performed, statistically significant differences between the curves were detected (sham vs. Ctrl-MSCs: F = 5.721, *p* = 0.0038; sham vs. GDNF-MSCs: F = 5.173, *p* = 0.0132; Ctrl-MSCs vs. GDNF-MSCs: F = 12.39, *p* < 0.0001; Fig. 3E). In the graphical representation, GNDF-MSCs seemed to have a positive effect in the first 2 weeks after transplantation (Fig. 3E1), whereas in the following 3 weeks, the curves were closer to those of the sham group (Fig. 3E2). Interestingly, however, the opposite effect was observed in the Ctrl-MSC group, where the cells seemed instead to halt seizure development in the last 3 weeks of the recording period (Fig. 3E2). In summary, Ctrl-MSC transplantation was demonstrated to have a beneficial effect on the development of SRSs, at least over the 35 days of video-EEG monitoring. In contrast, treatment with GDNF-MSCs seemed to have a therapeutic effect limited to the initial phase of epileptogenesis.

### Effects of hiAd-MSC transplantation on seizure frequency and inter-seizure intervals

**Fig. 4:**
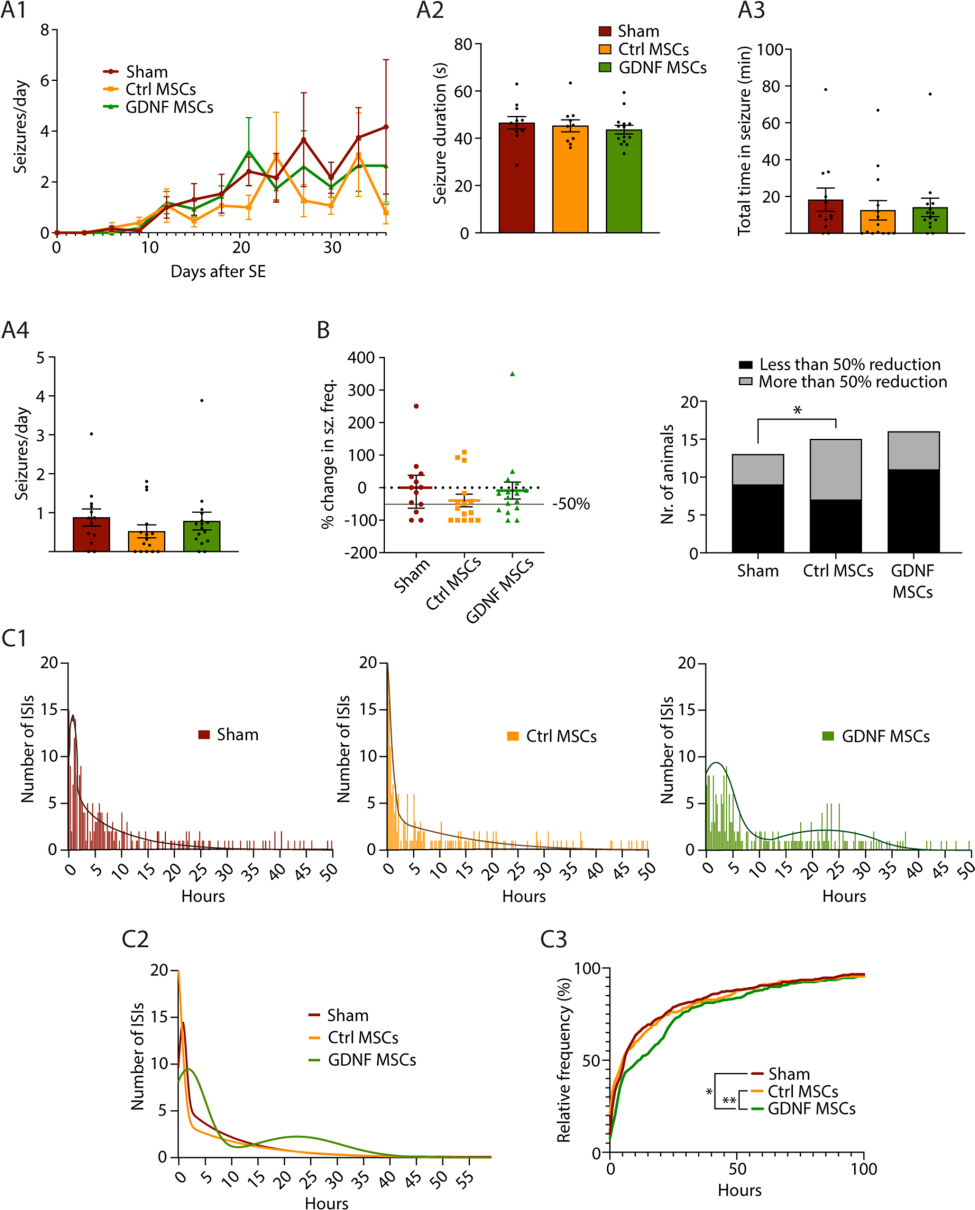
Effect of hiAd-MSC transplantation on seizure frequency. (**A1**) Seizure frequencies over time divided into 3-day bins were compared between the groups, and no significant differences were detected. (**A2**) The average seizure duration did not differ among the 3 groups. (**A3**) The total time spent in seizures did not differ between the groups. (**A4**) Seizure frequency, depicted as the average number of seizures per day, was assessed from the first detected seizure until the end of the monitoring. No differences were found. (**B**) The number of animals with a greater than 50% seizure frequency reduction compared with the median number in the sham group was compared between the groups. The proportions of these animals were significantly different between the sham and Ctrl-MSC groups. (**C1**) Interseizure interval distributions shown as histograms with fitted second-order Gaussian curves. (**C2**) Graphical comparison of the Gaussian curves of the 3 groups. (**C3**) The interseizure interval distribution, depicted as the relative frequency, differed among the 3 groups. Multiple Mann‒Whitney tests were used in A1. Kruskal‒Wallis test with Dunn’s multiple comparisons test was used in A2–A4. The c^2^ test was used for comparing proportions in B. Kolmogorov‒Smirnov test was used in C3. *, *p* < 05; **, *p* < 01

When analyzing SRS frequency (represented as the number of seizures per day, grouped into 3-day bins) over the 35 days of video-EEG recording, no differences among the treatment groups were found (Fig. 4A1). The average seizure duration was also not different between the groups (sham: 46.54 ± 2.6 s; Ctrl-MSCs: 45.26 ± 2.55 s; GDNF-MSCs: 43.62 ± 1.86 s; *p* = 0.3893; Fig. 4A2), nor was the total time spent in seizures (sham: 18.25 ± 6.32 min; Ctrl-MSCs: 12.49 ± 5.22 min; GDNF-MSCs: 14.06 ± 5.02; *p* = 0.4270; Fig. 4A3). Additionally, there was no difference in the overall seizure frequency among the 3 groups (sham: 0.88 ± 0.22 seizures/day; Ctrl-MSCs: 0.52 ± 0.17 seizures/day; GDNF-MSCs: 0.79 ± 0.23 seizures/day; *p* = 0.2049; Fig. 4A4). Changes in seizure frequency were also analyzed by comparing the average SRS frequency of each animal to the median frequency of the sham group, which was set as a baseline to calculate a percentage change for each animal. The significance level of change (to identify treatment responders) was set to a 50% reduction in seizure frequency compared with the sham median. This responder rate is a widely accepted benchmark for defining treatment response in epilepsy clinical research. It provides a clinically meaningful measure of therapeutic efficacy while allowing for comparisons across studies and interventions. The animals were then divided into those that reached this level and those that did not fulfill the criteria, and the proportions were then compared between the groups. A statistically significant difference was discovered when comparing the Sham and Ctrl-MSC groups, with more animals reaching the 50% seizure frequency reduction criterion when transplanted with Ctrl-MSCs (Sham: 4 out of 11 with > 50% reduction; Ctrl-MSCs: 8 out of 15 with > 50% reduction; *p* = 0.0350; Fig. 4B), but not when comparing the Sham group with GDNF-MSC-treated animals (5 out of 16 with > 50% reduction; *p* = 0.4047; Fig. 4B).

Next, we analyzed inter-seizure intervals (ISIs) and their distributions. The data are summarized in Fig. 3C, where distributions are represented as histograms and distribution-fitted curves for each group. In the histograms, a peak of short ISIs was detected in all 3 groups by fitting Gaussian curves (Fig. 4C1, C2). Notably, in the GDNF-MSC-treated group, a second peak was detected at 21.47 h, with the first peak having a lower number of events, indicating that in this group, there was a shift toward longer ISIs (Fig. 4C1, C2). No significant difference was observed in the relative distribution of the ISIs between the Sham and Ctrl-MSC groups (*p* = 0.0211, D = 0.1375). However, a significant difference in ISI distribution was detected between the sham and GDNF-MSC groups (*p* = 0.0024, D = 0.1593) and between the Ctrl-MSC and GDNF-MSC groups (*p* = 0.0001, D = 0.2018; Fig. 4C3). In summary, treatment with Ctrl-MSCs reduced SRS frequency compared with the median SRS frequency of the sham group, whereas GNDF-MSC-treated animals presented values similar to those of the sham animals. This finding is in line with the above-described effect on seizure occurrence (see Fig. 3). Interestingly, compared with the other 2 groups, the GNDF-MSC-treated group presented a change in ISI distribution, indicating that while total seizure numbers were not affected, GDNF release still prolonged the interval between individual seizures.

### hiAd-MSC transplantation partially restores epilepsy-related behavioral alterations

We next applied a battery of behavioral tests to identify the effects of cell transplantation on epilepsy-associated comorbidities, starting with the open field test for anxiety and locomotion analysis. This procedure was divided into 2 stages, both of which were 15 min long. In the first stage, anxiety variables were measured, whereas in the second stage, locomotion was examined. The main anxiety measure was based on the time spent in the center zone of the open-field arena. Anxious animals tend to spend less time exploring the center zone but prefer to be in proximity to walls. Group sizes and compositions were as follows: Control, *n* = 12; Sham, *n* = 15; Ctrl-MSCs, *n* = 15; GDNF-MSCs, *n* = 16.

**Fig. 5:**
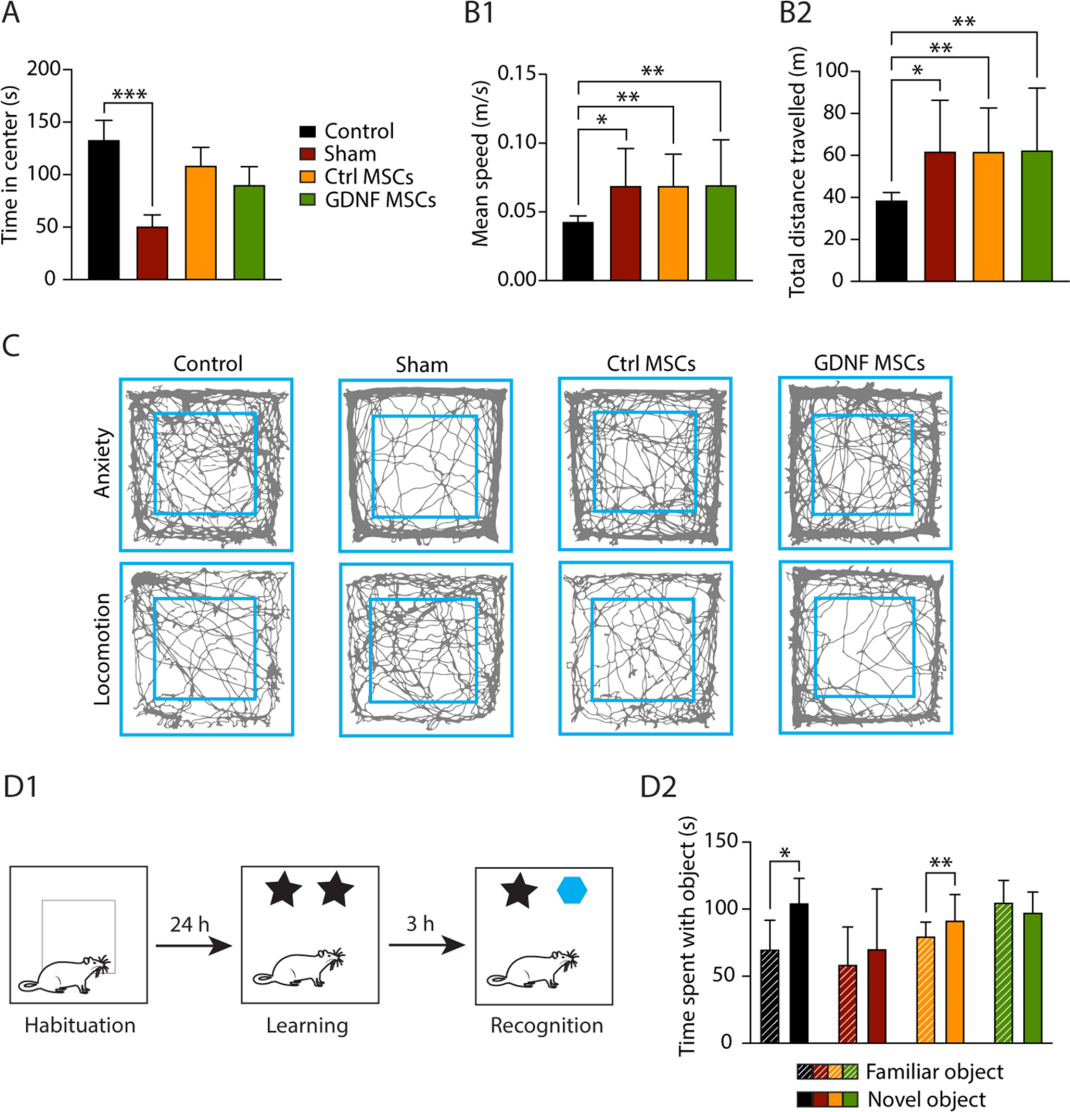
Effects of hiAd-MSCs on behavior assessed by open field and novel object recognition tests. (**A**) Anxiety levels represented the time spent in the center of the open field arena during the first 15 min of the test. Compared with healthy control animals, sham animals spent significantly less time in the center zone. No such differences were observed between the control, Ctrl-MSC-treated and GDNF-MSC-treated animals. (**B**) Locomotion measures, that is, the mean speed and total distance traveled, were assessed during the last 15 min of the open field test. All 3 experimental groups presented a greater mean speed than did the control group (**B1**) and a greater total distance traveled (**B2**). (**C**) Representative traces from both the anxiety and locomotion stages of the open field test for all 4 groups. (**D1**) Graphical illustration of the novel object recognition test. (**D2**) Control animals spent more time close to the novel object than did Ctrl-MSC-treated animals, whereas sham- and GNDF-MSC-treated animals failed to recognize the new object. Kruskal-Wallis test with Dunn’s multiple comparisons test was used in A and B, Wilcoxon test was used in D. *, *p* < 05; **, *p* < 01, ***, *p* < 001

The sham-transplanted epileptic animals presented higher anxiety levels than the healthy control animals did, represented by a significantly reduced time spent in the center of the arena (sham: 52.25 ± 4.66 s; control: 128.5 ± 8.85 s; *p* = 0.0006). No differences were detected between the healthy control and the Ctrl-MSC-treated animals (94.48 ± 14.06 s; *p* = 0.3530) or between the healthy control and the GDNF-MSC-treated group (98.82 ± 15.56 s; *p* = 0.1562; all the data are summarized in Fig. 5A). The assessment of average speed and total distance traveled revealed that both the sham- and MSC-treated animals (both Ctrl-MSC- and GDNF-MSC-transplanted) moved at a significantly greater mean speed than the healthy controls did (control: 0.042 ± 0.003 m/s; sham: 0.073 ± 0.007 m/s; Ctrl-MSCs: 0.078 ± 0.009 m/s; GDNF-MSCs: 0.075 ± 0.009 m/s; control vs. sham: *p* = 0.0131; control vs. Ctrl-MSCs: *p* = 0.0052; control vs. GDNF-MSCs: *p* = 0.0090; Fig. 5B1). The same outcome was observed when comparing the total distance traveled, where both the sham and MSC-treated animals covered a significantly longer distance than the healthy control animals did (control: 38.73 ± 3.04 m; sham: 65.42 ± 6.54 m; Ctrl-MSCs: 70.16 ± 8.32 m; GDNF-MSCs: 67.77 ± 7.76 m; control vs. sham: *p* = 0.0125; control vs. Ctrl-MSCs: *p* = 0.0059; control vs. GDNF-MSCs: *p* = 0.0010; Fig. 5B2). Representative traces from both stages of the open field test are depicted in Fig. 5C.

The novel object recognition test was used to examine short-term memory. A graphical scheme of the test is shown in Fig. 5D1. The time spent with each object was measured, and a comparison between the familiar and novel objects was made. The healthy control animals were able to successfully recognize the novel object, as shown by a statistically significant increase in time spent close to that object (familiar object: 75.58 ± 8.64 s; novel object: 106.20 ± 10.93 s; *p* = 0.0269), whereas the sham-transplanted epileptic animals could not differentiate between the novel and familiar objects (familiar: 72.95 ± 11.73 s; novel: 92.47 ± 11.30 s; *p* = 0.1531). Importantly, Ctrl-MSC-treated animals spent significantly more time close to the novel object, implying partial restoration of short-term memory by cell transplantation (familiar: 72.59 ± 5.91 s; novel: 100.60 ± 8.17 s; *p* = 0.0085). Interestingly, GDNF-MSC transplantation did not have the same effect, since these animals behaved more similarly to sham-transplanted animals (familiar: 102.10 ± 6.30 s; novel: 94.06 ± 6.63 s; *p* = 0.3028; data summarized in Fig. 5D2). In summary, both MSC lines exerted beneficial effects on anxiety behavior; however, they were not able to reverse epilepsy-related locomotor alterations. Notably, Ctrl-MSC but not GDNF-MSC transplantation restored the short-term memory deficit shown in sham-transplanted epileptic animals.

### hiAd-MSC transplantation does not influence microglial activation after 40 days

**Fig. 6:**
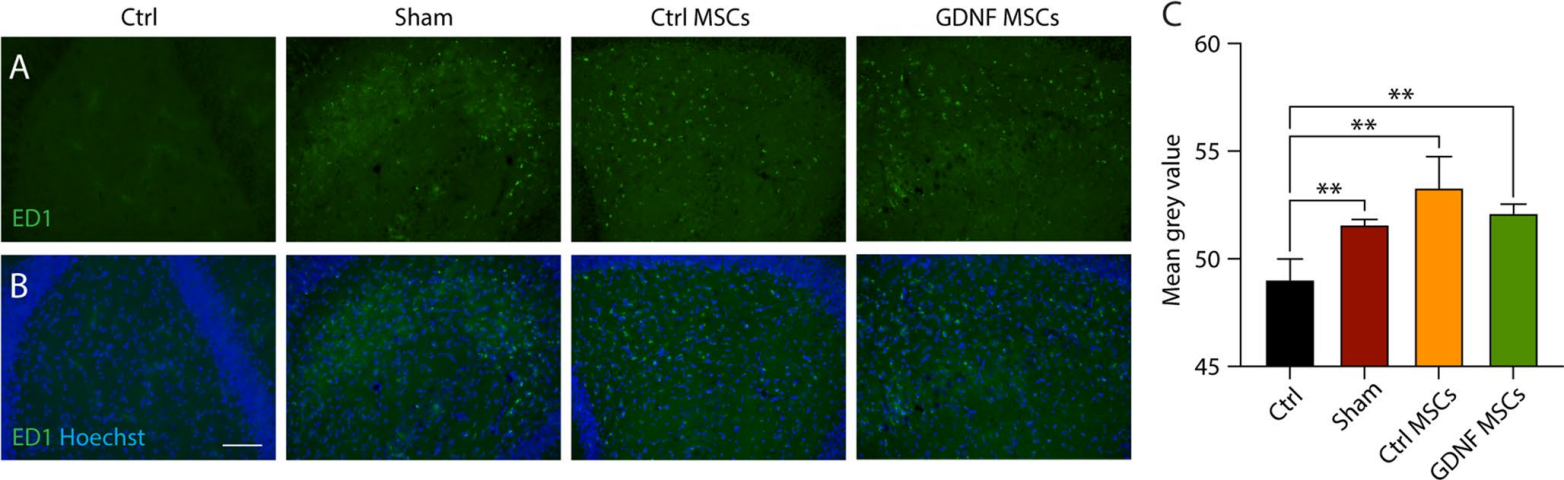
Microglial activation was assessed by staining for ED1 40 days after transplantation. (**A**) Representative images of ED1 (in green) staining in the dentate gyrus. (**B**) ED1 staining combined with cell nucleus staining (Hoechst, blue). (**C**) Quantification of the mean gray values for the 4 groups. Significantly higher values were observed in all the groups than in the healthy control group. The Kruskal‒Wallis test with Dunn’s multiple comparisons test was used in C. **, *p* < 01

To assess whether hiAd-MSC transplantation affects epileptogenesis-related inflammatory processes in the hippocampus, an immunohistochemical analysis of activated microglia was performed after 5 weeks of video-EEG monitoring and 2 days of behavioral tests (i.e., 40 days after cell transplantation). For the staining of activated microglia, ED1 (CD68) was used as a marker, and images from several hippocampal regions were analyzed (see methods section, representative images shown in Fig. 6A&B). Compared with the healthy controls, the sham animals had expectedly higher inflammation (activated microglia) levels in the hippocampus, which were, however, not ameliorated in any of the MSC-treated groups (control: 49.23 ± 0.24; Sham: 51.53 ± 0.14; Ctrl-MSCs: 53.85 ± 0.74; GDNF-MSCs: 50.73 ± 0.40; control vs. Sham: *p* = 0.0043; control vs. Ctrl-MSCs: *p* = 0.0043; control vs. GDNF-MSCs: *p* = 0.0065; data summarized in Fig. 6C). These data indicate that neither of the MSC treatments could counteract the activation of microglia, i.e., epilepsy-associated inflammation in the hippocampus, at the time of analysis. However, no remaining MSCs were observed within the tissue at this stage (stained for mCherry; images not shown), and whether changes in inflammation could be observed at the time when the cells were still alive (one or two weeks after transplantation) remains to be investigated.

## Discussion

Since many TLE patients do not respond to anti-seizure medications, it is necessary to develop new treatment strategies. In our study, we investigated the potential for early therapeutic intervention aimed at preventing the progression to the chronic phase of epilepsy. Specifically, we targeted the period immediately following the initial epileptogenesis-triggering event, which in our case was KA-induced SE. We transplanted either hiAD-MSCs or hiAD-MSCs engineered to release GDNF into the hippocampi of rats 16–24 h after SE induction to potentially modify the epileptogenesis process and prevent or reduce the severity and frequency of SRS as they began to manifest. We demonstrate that hiAd-MSC transplantation results in fewer animals developing SRSs, decreases SRS frequencies when they occur, diminishes anxiety levels, and restores short-term memory deficits. In contrast, GDNF-releasing hiAd-MSCs only reduced epilepsy-associated anxiety levels at 5 weeks after transplantation. In addition, the GDNF-MSCs seemed to decrease the probability of seizures in the first 2 weeks after SE. However, neither of the cell lines reduced inflammation levels in the epileptic hippocampus at the timepoint analyzed. In summary, hiAd-MSCs alone have been proven to improve epileptogenesis outcomes, whereas GDNF release from these cells has no significant additional beneficial therapeutic effect. As neither of the transplanted cell lines survived beyond the end of the observation period, this approach can be characterized as a form of transient cell therapy. These findings suggest that the therapeutic benefits observed were likely due to the temporary presence and activity of the cells rather than long-term cell integration.

The use of MSCs for the treatment of various disorders, including CNS conditions, has been extensively explored [15–18]. In addition to their proven therapeutic benefits, MSCs offer clinical advantages, such as the potential for autologous transplantation using patient-derived cells. These cells can be easily isolated from various tissue sources, expanded in culture, and cryopreserved if needed. Importantly, using patients’ own MSCs reduces the need for immunosuppression and avoids ethical concerns associated with other cell sources. Additionally, MSCs can serve as carriers for delivering therapeutic agents such as brain-derived neurotrophic factor [32], adenosine [10] or GDNF [33], which can provide further therapeutic benefits under diverse conditions. In epilepsy research, some animal studies have demonstrated the beneficial effects of MSC transplantation. While most studies have utilized rodent MSCs to influence the process of epileptogenesis [8–14], only a limited number have explored the therapeutic application of MSCs during the chronic phase of epilepsy [34, 35]. In our study, we aimed to evaluate the impact of *human MSCs* specifically on epileptogenesis, focusing on their immunomodulatory and neuroprotective effects during the latent phase [6] and shortly after the onset of the first seizures. Additionally, we investigated whether these cells could serve as effective delivery systems for GDNF, which has shown seizure-suppressing properties in various epilepsy models [25–28].

Our findings align with previous results from studies using a rat pilocarpine model of epilepsy, where intravenous treatment with rat bone marrow-derived MSCs (BMSCs) led to a reduction in the number of seizures occurring after SE during the 3-week video monitoring period (without EEG). Additionally, that study reported positive effects of BMSC treatment on neurodegeneration at 42 days post-SE. However, the study did not investigate the impact of the treatment on seizure activity beyond the initial 3-week period [9]. Similar effects of intravenous transplantation of rat BMSCs on epileptogenesis were observed in another study using a rat pilocarpine model. This study reported a reduction in seizure frequency, improved cognitive task performance, preservation of principal and inhibitory neurons in the hippocampus and suppression of aberrant mossy fiber sprouting. SRSs were counted from day 21 to day 30 post-SE, and only video monitoring was used for seizure detection. However, this method, which does not incorporate EEG recordings, likely underestimates the total number of seizures, as it does not capture non-convulsive seizures [12]. In another study with a rat pilocarpine model, rat BMSCs were transplanted intraventricularly 2 h after SE onset. EEG monitoring (without video recording) was conducted for 2 h per week from day 7 to day 28 post-SE to evaluate seizure activity. The authors reported a significant reduction in the number of EEG bursts in the BMSC-treated group compared with the sham control group [11]. However, the use of episodic recordings in this study might have introduced limitations, as seizure clustering reported in this epilepsy model could lead to underrepresentation of the total seizure burden [36–38]. In a different approach, Tamura and colleagues investigated the effects of intrahippocampal transplantation of mouse adipose tissue-derived MSCs on acute convulsive seizures. The MSCs were transplanted 10 days prior to inducing tonic‒clonic seizures via electroshock stimulation. Through visual monitoring, researchers reported that MSC-treated mice experienced shorter tonic‒clonic seizures and a lower mortality rate. Additionally, the study reported immunomodulatory effects of the treatment, as evidenced by altered gene expression of several cytokines [13]. Another study involving mouse MSCs focused on their protective effect on primary mouse neurons in vitro rather than their impact on seizures. This study demonstrated that MSCs reduce excitotoxic neuronal death caused by glutamate exposure and prevent the upregulation of NMDA receptor subunit expression. Interestingly, neurons treated with BMSCs presented an altered gene expression profile, which included the upregulation of stem cell and nonneuronal genes, indicating potential pathways through which MSCs exert their neuroprotective effects. To assess the effects of the same cells in vivo, mouse BMSCs were intravenously administered to the mice 24 h after systemic KA injection. Seven days later, the treatment resulted in reduced mossy fiber sprouting, decreased astrogliosis and decreased microglial activation [8]. It has also been demonstrated that MSC transplantation may have a restorative effect on the dysregulation of adenosine receptor expression associated with epilepsy. In this study, rat BMSCs were used for intrahippocampal transplantation in rats 1 month after pilocarpine-induced SE. This research is among the few that have investigated the potential of the use of MSCs for treating chronic epilepsy. The authors reported a positive effect of the treatment on epileptiform discharges observed 3 months posttransplantation. However, this conclusion was based on single, 15-minute-long EEG recordings [35], which may be subject to potential confounding factors such as seizure clustering. Another study employing rat BMSCs in the chronic phase of epilepsy using a rat pilocarpine model demonstrated that 15 days after transplantation, treated animals presented normalized levels of both excitatory and inhibitory neurotransmitters in the brain, along with a reduction in oxidative stress markers, proinflammatory cytokines, and neurodegeneration. The authors do not report any effect on SRSs. However, they compared the two preferred routes of cell administration: intravenous and intrahippocampal. According to their findings, intrahippocampal BMSC transplantation led to greater cell integration with the hippocampus and achieved a superior therapeutic outcome [34].

The application of human MSCs for transplantation in epilepsy models remains relatively limited. In a study by Huang and colleagues, human umbilical cord-derived MSCs were transplanted into the hippocampus of rats 1 day after pilocarpine-induced SE [14]. Video-EEG monitoring was conducted from 2 to 4 weeks post-SE, with a recording duration of only 9 h per week, revealing a reduction in SRSs and a shorter SRS duration in MSC-treated rats. Additionally, MSC transplantation was associated with reduced brain edema, decreased neuronal loss, and attenuation of neuroglia activation in the hippocampus at 29 days post-SE. The study also demonstrated a neuroprotective effect of MSCs in vitro, showing that they could prevent glutamate-induced cytotoxicity [14]. However, the episodic nature of seizure monitoring presents a potential limitation of this study. In a study by Li and colleagues, human bone marrow-derived MSCs engineered to release adenosine were transplanted into the hippocampi of mice 24 h after SE was induced by intra-amygdaloid injection of KA. Continuous 24-hour EEG monitoring, conducted 3 weeks posttransplantation, revealed a reduction in electrographic seizure frequency and duration in the treated group. However, the authors attributed this effect to adenosine release rather than MSC transplantation per se, as the positive effects were counteracted by an adenosine receptor antagonist. Notably, this study was conducted under cyclosporine-mediated immunosuppression, which may enhance cell survival but could compromise the inherent immunomodulatory effects of MSCs [10]. This study aligns with previous findings by the same authors, who demonstrated the beneficial effects of adenosine-releasing human MSCs on acute seizures [39].

Human umbilical cord-derived MSCs have been shown to survive in the rat hippocampus for up to 4 weeks post-transplantation without requiring immunosuppression [14]. However, their survival beyond 30 days in the rat brain has not been demonstrated [40]. In our study, we observed MSC survival at 7 and 14 days after transplantation but not after 21 or 40 days, likely due to immune rejection of human cells in the rat brain [41]. We leveraged this limited survival period of human MSCs in the rat hippocampus to examine the effects of transient cell therapy on epileptogenesis and the early chronic phase, during which seizures occur. Although MSCs did not persist until the study’s endpoint, we observed positive outcomes of such short-term transient interventions. Previous studies have reported that MSCs exhibit neuroprotective effects and inhibit astrogliosis and microglial activation in a mouse KA-SE model at 7 days post-transplantation [8]. These early post-transplantation effects of MSCs on inflammatory processes may be sufficient to protect against seizure occurrence, as we observed no sustained impact on microglia activation at the later 40-day timepoint. This lack of lasting effect could be attributed to the limited survival of the transplanted cells up to this timepoint.

Notably, in all previously mentioned studies involving both rodent and human MSCs, the progression of SRS occurrence over time was not thoroughly examined. Both effects on seizures were not investigated, or only brief periods of seizure recording were analyzed. In our study, however, we employed continuous 24/7 video-EEG monitoring for 35 days, beginning immediately after cell transplantation. This approach allowed us to capture electrographic and behavioral seizure activity throughout the entire epileptogenesis process and, crucially, to correlate late-stage seizures with video confirmation. By excluding potential false positives due to seizure clustering [42, 43], our results provide a more reliable and conclusive assessment of treatment effects.

Our data indicate that GDNF-MSC transplantation yielded fewer beneficial effects than did naïve MSC transplantation. However, our results suggest that GDNF-MSCs may induce a partial shift toward longer ISIs and exert an inhibitory effect on seizure occurrence during the first 2 weeks after SE induction. In support of our findings, previous studies have shown that blocking GDNF signaling following intrahippocampal KA-SE in mice led to a more severe epileptogenic outcome, characterized by increased neurodegeneration and earlier onset of SRSs, underscoring the beneficial role of GDNF during epileptogenesis [29]. In our previous study, we demonstrated that GDNF overexpression through rAAV vector injection in the rat hippocampus effectively suppressed the induction of acute generalized seizures. Additionally, we observed a GDNF-mediated increase in the seizure threshold when the treatment was introduced in electrically kindled animals [25]. Our work also revealed that semipermeable capsules containing GDNF-releasing cells placed in the rat hippocampus attenuated seizures following repeated electrical kindling [26]. Furthermore, a seizure-suppressing effect was observed when encapsulated GDNF-releasing cells were implanted unilaterally into the hippocampus of rats that had undergone intrahippocampal KA-induced SE 10 weeks earlier [27]. In this study, using continuous long-term video-EEG monitoring for seizure detection, we reported a reduction in SRS frequency and cumulative seizure count following GDNF treatment [27]. The beneficial effects of GDNF-releasing cells have also been observed in other epilepsy models [28]. In the rat pilocarpine SE model, for example, encapsulated GDNF-releasing cells were implanted bilaterally during the chronic stage of epilepsy, resulting in a significant reduction in SRS frequency, prevention of hippocampal volume loss, and decreased neurodegeneration [28]. These findings collectively support the potential of GDNF as a therapeutic approach in various epilepsy contexts.

Compared to these previous strategies for GDNF delivery, such as recombinant AAV (rAAV)-mediated overexpression [25] or encapsulated cell implants [26–28], our approach using genetically modified MSCs presents both distinct advantages and limitations. rAAV-GDNF delivery has shown robust and long-lasting seizure suppression but requires permanent transgene expression and poses translational challenges related to vector immunogenicity and long-term safety. Similarly, encapsulated GDNF-secreting cells have demonstrated efficacy in multiple epilepsy models, particularly in chronic stages, with sustained GDNF release and neuroprotection. However, these devices require invasive implantation and long-term viability in the host brain.

In contrast, our approach is characterized by transient, cell-based GDNF delivery during early epileptogenesis using human MSCs, which were not detectable beyond 21 days post-transplantation. Despite this, we observed a partial seizure-suppressing effect in the early phase after SE, along with a shift in inter-seizure interval distribution. This suggests that even short-term GDNF delivery via MSCs may confer temporally limited but potentially clinically relevant benefits. Additionally, our strategy combines the inherent immunomodulatory and neuroprotective effects of MSCs with localized GDNF expression, providing a multifactorial intervention that could be tailored to different stages of epileptogenesis.

Importantly, while prior GDNF studies primarily focused on chronic epilepsy models, our study uniquely targets the latent phase immediately after SE, a critical window for modifying disease progression. Thus, our findings position MSCs as a flexible, transient delivery platform for neurotrophic factors, offering a minimally invasive and time-restricted alternative to gene therapy or encapsulated devices, particularly relevant for early intervention strategies.

Recent studies have shown that both dimethyl fumarate, through activation of the Nrf2 antioxidant pathway [44], and trilostane, via enhancement of endogenous neurosteroids and modulation of neuroinflammation [45], exert disease-modifying and antiepileptogenic effects in preclinical epilepsy models. In comparison, our approach using GDNF-secreting MSCs offers a localized, cell-based intervention that combines neurotrophic support with broad paracrine modulation of inflammatory and excitotoxic pathways during the latent phase, providing an alternative strategy for targeting epileptogenesis at an early stage.

However, a key limitation of our study is the transient survival of the transplanted human MSCs, which were no longer detectable beyond 21 days post-transplantation. Consequently, the observed therapeutic effects, such as reduced seizure burden and partial behavioral recovery, are most likely mediated by acute paracrine signaling rather than long-term integration or sustained neurotrophic support. This distinction is important for understanding the underlying mechanisms, as the interventions may have acted by modulating early inflammatory or excitotoxic cascades during epileptogenesis, rather than inducing durable structural changes in epileptic networks.

For clinical translation, strategies to prolong MSC survival, use autologous or immune-compatible sources, or incorporate adjunctive treatments may enhance efficacy. Nevertheless, the transient nature of MSC-based interventions may still be advantageous, particularly in future clinical contexts where reliable stratification biomarkers can identify individuals at high risk of developing epilepsy following seizure-precipitating events. In such cases, timely and short-term cell-based therapies could be deployed during the latent phase to modify disease progression without the need for long-term graft survival.

An important consideration for future translational development of MSC-based therapies is their potential interaction with concurrently administered antiseizure medications (ASMs). As reviewed by [46], several commonly used ASMs can influence key properties of mesenchymal stem cells, including their survival, differentiation capacity, and paracrine factor release. Given that most patients at risk of developing epilepsy or undergoing early interventions are likely to be treated with ASMs, it will be essential to assess how such medications may modulate the therapeutic efficacy of transplanted MSCs. Future studies should therefore include systematic evaluation of drug-cell interactions in both in vitro and in vivo settings to optimize the clinical applicability of MSC-based approaches for epilepsy prevention or treatment.

## Conclusion

Our study provides compelling evidence that reinforces and expands the growing body of research demonstrating the therapeutic benefits of MSC transplantation in mitigating epileptogenesis. These new insights into the therapeutic potential of MSCs, whether with or without GDNF release, highlight their promise for developing novel cell therapies targeting the epileptogenesis process. This approach opens exciting avenues for future clinical translation, advancing the potential for cell-based interventions in epilepsy treatment.

## Supplementary Information

Below is the link to the electronic supplementary material.

Supplementary Material 1

## Data Availability

The datasets used and/or analysed during the current study are available from the corresponding author on reasonable request.
